# Genetic reporter analysis reveals an expandable reservoir of OCT4+ cells in adult skin

**DOI:** 10.1186/2045-9769-3-9

**Published:** 2014-06-14

**Authors:** Anne Limbourg, Sabine Schnabel, Vladimir J Lozanovski, L Christian Napp, Teng-Cheong Ha, Tobias Maetzig, Johann Bauersachs, Hassan Y Naim, Axel Schambach, Florian P Limbourg

**Affiliations:** 1 Research Group Regenerative Agents, Hannover, Germany; 2 REBIRTH Cluster of Excellence, Hannover, Germany; 3 Integrated Research Center Transplantation (IFB-Tx), Hannover, Germany; 4 Present Address: Department of Plastic, Hand and Reconstructive Surgery, Hannover, Germany; 5 Department of Cardiology and Angiology, Hannover, Germany; 6 Institute of Experimental Hematology, OE6960 Hannover Medical School, Carl-Neuberg-Str. 1, D-30625 Hannover, Germany; 7 Department of Physiological Chemistry, Hannover Veterinary School, Hannover, Germany; 8 Division of Hematology/Oncology, Boston Children’s Hospital, Harvard Medical School, Harvard, USA; 9 Department of Nephrology and Hypertension, Hannover Medical School, Hannover, Germany; 10 Present Address: Department of General and Transplant Surgery, University Hospital Heidelberg, Heidelberg, Germany; 11 Vascular Medicine and Transplantation Research, Dept. of Nephrology and Hypertension, OE 6841, Hannover Medical School, Carl-Neuberg-Str. 1, D-30625 Hannover, Germany

**Keywords:** Oct4, Epidermal stem cells, Skin, Transgenic reporter mice

## Abstract

The transcription factor *Oct4* (*Pou5f1*) is a critical regulator of pluripotency in embryonic and induced pluripotent stem cells. Therefore, *Oct4* expression might identify somatic stem cell populations with inherent multipotent potential or a propensity for facilitated reprogramming. However, analysis of *Oct4* expression is confounded by *Oct4* pseudogenes or non-pluripotency-related isoforms. Systematic analysis of a transgenic *Oct4-EGFP* reporter mouse identified testis and skin as two principle sources of *Oct4* ^+^ cells in postnatal mice. While the prevalence of GFP^+^ cells in testis rapidly declined with age, the skin-resident GFP^+^ population expanded in a cyclical fashion. These cells were identified as epidermal stem cells dwelling in the stem cell niche of the hair follicle, which endogenously expressed all principle reprogramming factors at low levels. Interestingly, skin wounding or non-traumatic hair removal robustly expanded the GFP^+^ epidermal cell pool not only locally, but also in uninjured skin areas, demonstrating the existence of a systemic response. Thus, the epithelial stem cell niche of the hair follicle harbors an expandable pool of Oct4+ stem cells, which might be useful for therapeutic cell transfer or facilitated reprogramming.

**Electronic supplementary material:**

The online version of this article (doi: 10.1186/2045-9769-3-9) contains supplementary material, which is available to authorized users.

## Background

Regenerative cell therapy for terminally differentiated organs damaged by disease requires multipotent stem cell reservoirs, preferably from autologous sources. The POU-domain transcription factor *Oct4* (*Pou5f1*) is an important regulator of pluripotency in embryonic stem (ES) and induced pluripotent stem (iPS) cells [1]. The latter arise during re-programming from clones re-expressing endogenous *Oct4* [2], while under specific culture conditions ectopic expression of *Oct4* alone in primary somatic cells is sufficient for reprogramming into iPS or multilineage progenitors [3, 4]. Therefore, endogenous *Oct4*+ stem cells might either display inherent multipotent differentiation potential or serve as a source for minimal modification approaches to generate iPS with high efficiency. However, analysis of *Oct4* expression is confounded by false-positive results due to *Oct4* pseudogenes or non-pluripotency-related, non-nuclear OCT4B isoforms, explaining contradictory reports on *Oct4* expression in several somatic stem cell populations [5].

The hair follicle, as part of the protective and self-renewing mammalian epidermis, is one of the few organs that undergoes constant cycles of degeneration and regeneration throughout life. It contains an epidermal stem cell niche harboring multipotent epidermal stem cells, which can be mobilized to regenerate the new follicle with each hair cycle or in damaged skin during wound repair [6]. Multipotent epidermal stem cells not only give rise to new epidermis and hair when grafted but are able to correct inherited skin disease in humans and differentiate into all principle tissue lineages in culture [6–9], while a population of skin stem cells can contribute to skeletal muscle fiber regeneration in muscle dystrophy after cell transplantation [10]. Furthermore, a poorly characterized subset of cells isolated from human hair follicles has been shown to express *Oct4* and to display multipotent behavior in vitro [11]. Our objective was to identify adult somatic stem cells expressing *Oct4* using a well characterized Oct4-GFP genetic reporter mouse model as a potential reservoir for regenerative cell therapy.

## Results

To identify in a genetic screening approach OCT4^+^ stem cells during postnatal life, we analyzed different tissues of Pou5f1-EGFP transgenic reporter mice (called OG2) for GFP expression by flow cytometry. These mice express enhanced GFP under the control of the *Oct4* promoter including the distal enhancer, and expression of GFP has been shown to serve as well described reporter for endogenous, pluripotency-related OCT4 expression in induced pluripotent stem cells as well as during development and more interestingly postnatal life [12–15].

**Figure 1:**
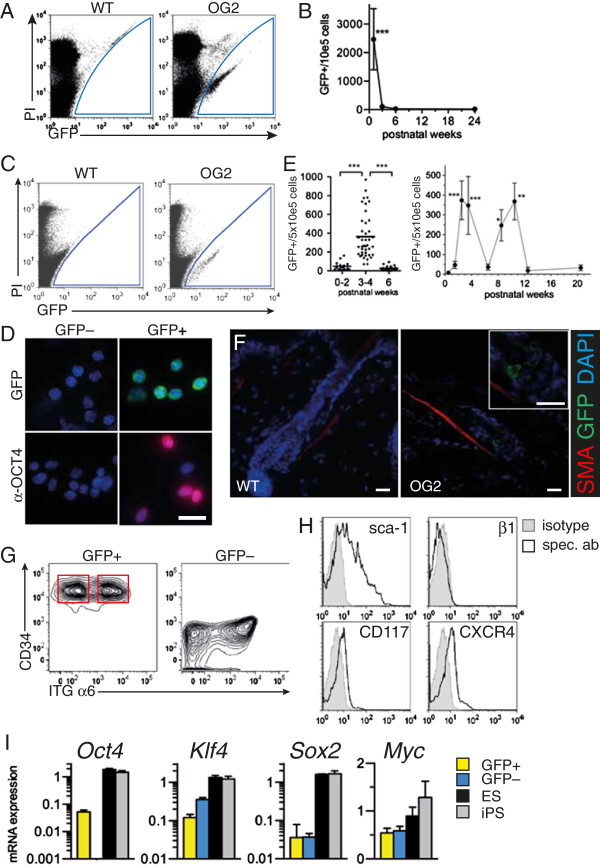
**Characterization of GFP** ^**+**^ **cell populations in testis and skin in Oct4-Gfp (OG2) reporter mice. (A)** Flow cytometry of cell suspensions from 1 wk old neonatal testis with compensation for autofluorescence by plotting FL1 against FL2. Dead cells are stained with propidium iodide (PI). **(B)** Timecourse analysis by flow cytometry of GFP + cells in testis at 0–2 wks, 3–4 wks, 6 wks and 24 wks of age. P vs 0–2 wks. **(C)** Representative FACS analysis and GFP^+^ gating of cell suspensions from skin. **(D)** Fluorescence microscopy of sorted GFP^+^ and GFP^-^ cells for GFP, anti-OCT4 antibody staining (red) and nuclear dye (DAPI, blue). **(E)** GFP^+^ skin cells by FACS in different age groups (left), n = 36-45. Cyclic expansion of the GFP^+^ cell pool over time (right). n = 9-30. P vs 0.5 wks. **(F)** Immunostaining of hair follicle bulge region against smooth muscle α–actin (SMA) and GFP. Insert from same image in higher magnification. **(G)** Flow cytometry of GFP+ or GFP- gated cells from skin. CD34^+^ ITGα6^hi^ and CD34^+^ ITGα6^lo^ populations are indicated. **(H)** Representative flow cytometry profile of GFP^+^ gated cells stained with specific antibodies (spec. ab) or isotype control (isotype). **(I)** mRNA expression (2^ΔCt^) by quantitative real-time PCR of pluripotency factors from sorted skin cells or cultures of iPS and ES cells. n = 3-4. Scale bar: 20 um. Magnification: **(D)** 640, **(F)** 200, 640 (insert).

We verified the fidelity of the reporter by analyzing postnatal testis, since a subset of male spermatogonial stem cells (SSC) have been shown to express OCT4 and can give rise to pluripotent stem cells [12, 16–18]. FACS analysis of newborn mouse testis revealed a distinct cell population with specific GFP-fluorescence expressing OCT4 (Figure 1A, Additional file 1: Figure S1, Table 1). However, the prevalence of GFP^+^ cells in testis was highest in the early postnatal period but rapidly declined to permanently low levels with maturation ( ≥3-4 wks, Figure1B).

**Table 1:** **Frequency of GFP + cells in different mouse tissues and age groups**

Organ	Age	GFP+/500.000 cells			Analyzed cells/mouse
		mean	SD	n	mean
Heart	Infant	0	0	12	3.58E + 06
	Puberty	0	0	15	2.83E + 07
	Adult	0	0	14	2.46E + 07
Heart p MI	12 wks	0	0	8	2.67E + 07
Bone marrow	Infant	0	0	11	2.49E + 07
	Puberty	0	0	14	4.22E + 07
	Adult	0	0	10	5.87E + 07
Spleen	Infant	0	0	14	2.83E + 07
	Puberty	0	0	10	8.97E + 07
	Adult	0	0	9	7.03E + 07
Testis	Infant	2791	1229	9	1.22E + 06
	Puberty	110	77	11	3.00E + 07
	Adult	27	20	15	1.52E + 07
Skin	Infant	50	39	25	8.30E + 06
	Puberty	396	245	23	6.35E + 06
	Adult	31	25	31	7.57E + 06

Infant: 1–2 wks, puberty: 3–4 wks, adult: 6 wks, hearts p MI (post myocardial infarction) were harvested between d3 and d7.

Screening of various tissues reported to harbor *Oct4* expressing stem cells, such as heart and bone marrow, did not detect GFP^+^ cells in these tissues, although large cell numbers from each organ were analyzed (Table 1). As Oct4 expression was also found in human adipose tissue derived stem cells [19], we also analyzed GFP expression in the inguinal adipose tissue, but could not detect GFP^+^ cells in 4 analyzed animals (data not shown). However, GFP^+^ cells were discovered in the skin (Table 1, Figure 1C), which express the nuclear, pluripotency-related OCT4 isoform (Figure 1D). This cell population expanded transiently and in a biphasic fashion during postnatal life, peaking at puberty and early adulthood, which was reminiscent of the synchronized and cyclic activity of the hair follicle during the first hair cycles (Figure 1E). In situ, GFP^+^ cells localized to the bulge region of the hair follicle, which is a niche for multipotent epithelial stem cells and marked by the insertion of smooth muscle cells (Figure 1F) [6]. These multipotent stem cells consist of a basal and suprabasal population, which behave analogously with respect to self-renewal and differentiation potential, and which are defined by expression of CD34 and high or low expression of integrin (ITG) α6, respectively [6]. Flow cytometry of GFP^+^ and GFP^-^ skin cells revealed that GFP^+^ cells were found in both stem cell subsets, but not in the non-stem cell compartment (Figure 1G). These data suggest that GFP expression identifies an *Oct4* ^*+*^ population of multipotent epidermal stem cells residing in the stem cell niche of the hair follicle. Further characterization of the surface marker profile of GFP^+^ stem cells revealed a phenotype of Sca-1^+^, CD117^lo^, CXCR4^lo^ and ITG β1^-^ (Figure 1H). In contrast to GFP- cells, GFP + cells also showed evidence for Oct4 mRNA expression, as demonstrated by primers specific for exon 1, but expression levels were considerably lower than in ES or iPS controls. Interestingly, GFP^+^ cells also expressed low amounts of other key iPS reprogramming factors, *Klf4, Sox2* and *c-Myc,* however, these were also present in GFP- cells (Figure 1I).

**Figure 2:**
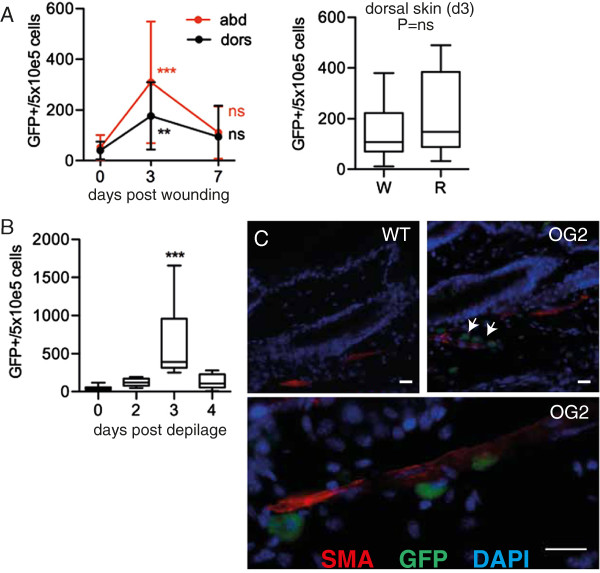
**Dynamic expansion of GFP** ^***+***^ **cells in skin. (A)** Localized punch injury to dorsal skin. Left, FACS count of GFP^+^ cells from dorsal (dors) and abdominal (abd) skin. n = 14-28. P vs. same side basal (0). Right, box blot of epidermal GFP^+^ cells from wound area (W) vs remote (R) dorsal skin at d3. n = 13. **(B)** Box plot of epidermal GFP^+^ cells by FACS after non-traumatic skin depilation, age 12–14 wks, n = 9-28. P vs. basal (d0). **(C)** Immunostaining against smooth muscle–actin (red) and GFP (green) in flank skin after dorsal depilation, DAPI (blue). Scale bar: 20 um, magnification: 400 (upper panel), 640 (lower panel). GFP- gated cells from skin. CD34^+^ ITGα6^hi^ and CD34^+^ ITGα6^lo^ populations are indicated. (H).

The cyclic and synchronized expansion of GFP^+^ skin cells observed during postnatal maturation suggested regulation by external cues. To test this hypothesis we analyzed the behavior of GFP + cells following dorsal skin wounding by punch biopsy in adult mice. Follwing wounding, there was a rapid and transient increase in GFP^+^ cells in dorsal skin. However, this response was not limited to areas of injury, but also occurred in remote dorsal skin areas, and even in abdominal skin (Figure 2A). This suggests a systemic signal controlling GFP^+^ cell expansion in response to injury. Furthermore, non-traumatic hair removal by depilation, which stimulates the hair follicle without skin injury, induced a comparable expansion of stem cells throughout the skin (Figure 2B, and data not shown). Following depilation, GFP^+^ cells were also detected outside the bulge region along the structures provided by smooth muscle cells, suggesting mobilization (Figure 2C).

## Discussion

Using an *Oct4-GFP* transgenic reporter, we identify skin as a novel principle source of postnatal Oct4^+^ cells. We also confirm previous reports on the existence of OCT4+ cells in neonatal testis [12, 16–18]. However, numbers of GFP^+^ cells in testis decline rapidly and permanently before puberty. In contrast, the skin-resident cell pool expands postnatally in a cyclic fashion strikingly matching the time-course of the cyclic hair follicle activity during the first postnatal hair cycles, which in the mouse occurs in a synchronized fashion [20]. In situ, GFP^*+*^ cells localize into the bulge region of the hair follicle, which constitutes a niche for two populations of multipotent epidermal stem cells. Surface marker analysis demonstrates that GPF + cells are present in both stem cell subsets. Furthermore, the adult GFP^*+*^ stem cell pool expands in the entire skin in response to injury or non-traumatic depilation, suggesting so far unknown systemic signals controlling stem cell expansion. This also establishes a simple but defined setting for cell expansion for therapeutic cell harvest, i.e. depilation. The here characterized *Oct4* ^*+*^ epidermal stem cells not only express the nuclear OCT4 isoform but – albeit at very low levels – other factors of the reprogramming cocktail. Notably, epidermal stem cell populations with similar characteristics also exist in humans [11, 21]. Thus, skin might serve as a reservoir for an expandable stem cell population, which could be useful for therapeutic cell transfer or facilitated reprogramming to generate iPS ex vivo.

The functional relevance of *Oct4* expression, and the biological potential of the identified cell population, requires further studies. Mice with conditional-inducible deletion of *Oct4* in skin cells using the keratin K15 promoter showed normal hair follicle architecture and skin wound healing response in short-term experiments [22]. However, the long-term consequences of *Oct4* deletion were not studied. Furthermore, multipotent epidermal stem cells in the hair follicle bulge have been identified and characterized based on K14 expression [6], leaving open the possibility of an escape from *Oct4* inactivation in this study.

Of note, Dyce and colleagues have differentiated skin-derived stem cells into oocyte-like cells, i.e. Oct4^high^ germ cells [23]. In light of our findings and recent reports that culture conditions strongly influence cell fate control, e.g. as for gPS, one could envision that Oct4^+^ skin cells have been reprogrammed under appropriate culture conditions into Oct4^high^ germ cells, e.g. oocyte-like cells.

Importantly, just recently human multipotent dermal stem cells have been isolated based on neural crest stem cell marker nestin [24]. Importantly, these cells were also positive for Oct4, suggesting that Oct4 positivity of dermal stem cells might be conserved across species.

## Conclusions

Our findings of an expandable Oct4+ stem cell pool in the skin may have implications for facilitated reprogramming or for the development of new skin stem cell-based therapeutic models. In addition, if it is possible to culture Oct4+ epithelial stem cells *in vitro*, this will also ease genetic modification of skin stem cells to repair genetic defects, e.g. for gene therapy of epidermolysis bullosa.

## Methods

### Antibodies

Rabbit anti-Oct4 sc-9081 (Santa Cruz), mouse anti-SMA-Cy3 C6198 (Sigma), rabbit anti-GFP SP3005P (Acris), rat anti-c-kit/CD117 clone 3C1 (Miltenyi Biotec), rat anti-Cxcr4 FAB21651A (R&D Systems), rat anti-sca-1 clone D7 (Miltenyi Biotec), rat anti-α6-Integrin clone GH3 (R&D Systems), rat anti-CD34, clone MEC14.7 (Biolegend).

### Primers

*RT-PCR*: *Oct4*, fwd: gcgttctctttggaaaggt, rev: agcctcatactcttctcgttgg. Rex-1, fwd: caccatccgggatgaaagtgagat, rev: accagaaaatgtcgctttagtttc. GAPDH: fwd: accacagtccatgccatcac, rev: tccaccaccctgttgctgta. *Real-time PCR*: *Oct4*, fwd: gttggagaaggtggaaccaa, rev: ctccttctgcagggctttc. *Myc*, fwd: cgcctagaattggcagaaat, rev: aactgagaagaatcctattcagcac. *Klf4*, fwd: cgggaagggagaagacact, rev: gagttcctcacgccaacg. *Sox2*, fwd: tgctgcctctttaagactaggg, rev: tcgggctccaaacttctct. Quantitative real-time PCR was carried out on the LightCycler 480 System (Roche).

### Mice

The study was conducted with permission of the State of Niedersachsen, conforming to the German Law for the Protection of Animals and the NIH *Guide for the Care and Use of Laboratory Animals*. Pou5f1-egfp reporter mice (B6; CBA-Tg (Pou5f1-EGFP) 2Mnn), have been described before and were housed at SPF conditions [12].

### Cell isolation and FACS analysis

Tissues were dispersed mechanically or digested with Collagenase I (0.25% w/v) and DNAse I (360 U/ml) for 45 min at 37 C. For isolation of skin-resident cells, mice were shaved thoroughly and abdominal, dorsal and flank skin carefully excised. Whole skin was spanned with the epidermis facing down and dermal fat mechanically removed using curved tweezers. Skin was washed in PBS, finely homogenized using surgical scalpels and digested. The released cells where recovered by centrifugation, washed several times with PBS with 2% FCS, stained with propium iodide solution and analyzed on a FACSCalibur (BD Bioscience). Specific GFP-fluorescence was discriminated from autofluorescence and dead cells by plotting FL-1 vs FL-2 with uncompensated settings.

### Statistics

Data are given as mean and SEM, unless stated otherwise. Comparisons were performed by Student’s t-test or ANOVA with Bonferroni’s or Dunnett’s post-test.

## Electronic supplementary material

Additional file 1: Figure S1: Co-expression of GFP and nuclear OCT4 in neonatal testicular cells. Fluorescence microscopy, native green fluorescence (GFP) in cells showing nuclear anti-OCT4 staining. Scale bar: 20 um. (PDF 148 KB)

## Abbreviations

ES: Embryonic stem cell
iPS: Induced pluripotent stem cells
K14: Cytokeratin-14
K15: Cytokeratin-15
FACS: Fluorescent activated cell sorter
SD: Standard deviation
SPF: Specific pathogen free
GFP: Green fluorescent protein
FL: Fluorescence
ITG: Integrin
PBS: Phosphate-buffered saline
FCS: Fetal calf serum
gPS: Germline-derived pluripotent stem cells.
