# Supplementary figures and images for: Genetic reporter analysis reveals an expandable reservoir of OCT4+ cells in adult skin
Source: Cell Regen. 2014 Jun 14;3:9. doi: 10.1186/2045-9769-3-9 (PMC4230759; doi:10.1186/2045-9769-3-9)

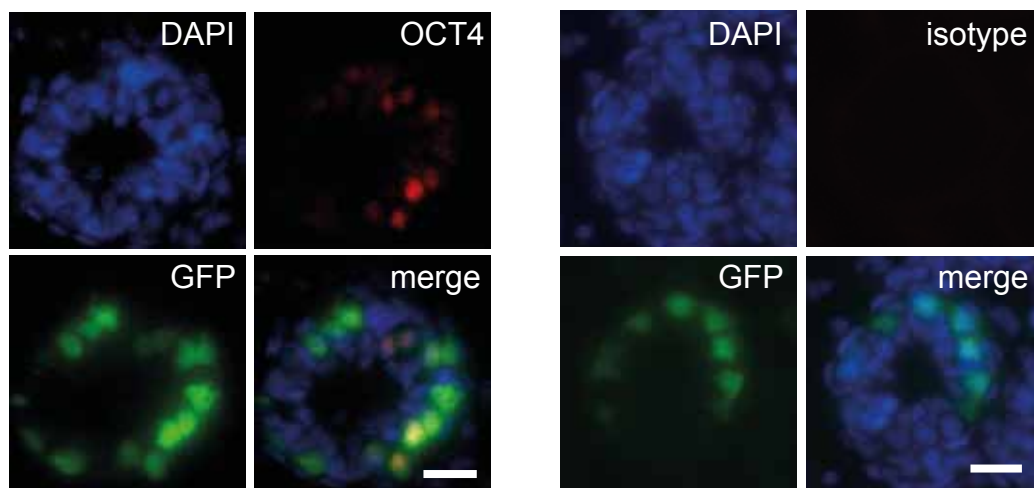

Supplement: Supplementary file 1 — Additional file 1: Figure S1: Co-expression of GFP and nuclear OCT4 in neonatal testicular cells. Fluorescence microscopy, native green fluorescence (GFP) in cells showing nuclear anti-OCT4 staining. Scale bar: 20 um. (PDF 148 KB) [file 13619_2013_25_MOESM1_ESM.pdf]
